# Supplementary material for: Analysis of sensorineural hearing loss in patients attending an otolaryngology clinic in North Central Nigeria
Source: PLOS Glob Public Health. 2023 Apr 25;3(4):e0000685. doi: 10.1371/journal.pgph.0000685 (PMC10128921; doi:10.1371/journal.pgph.0000685)
Supplement: Attachment — Submitted filename: PLOSGPHRevisionLetter060422.docx [file pgph.0000685.s002.docx]

June 5, 2022

Re: PGPH-D-21-00966

Dear Dr. Moroe,

Thank you for the opportunity to submit a revised draft of our manuscript entitled, "A ten-year retrospective review of audiogram configurations of patients attending an otolaryngology clinic in Lassa fever endemic North Central Nigeria. '' to the *PLOS Global Public Health* Journal. We appreciate the effort and time that the reviewers dedicated in providing their insight and helpful feedback. We have revised the manuscript incorporating the changes from the suggestions that were provided. We hope our responses address the concerns the reviewers have provided and have improved the quality of the manuscript.

The following is a point-by-point response to the questions and comments.

**REVIEWER #1:**

1. METHODOLOGY
   a. The authors state, “Specifically, the study assessed the burden of moderate or higher degree hearing loss at different age groups…” but the method does not delineate how burden is measured since it is not only an audiometric concept. Moreover, the aims could be specifically detailed.

**RESPONSE:** The authors appreciate the input and agree with the need for more clarity. We were trying to convey that the study assesses prevalence rather than burden. We have revised the Introduction section as follows for clarity.

“The overall goal of this study was to determine the period prevalence of and patterns of audiograms of patients attending an otolaryngology clinic in North Central Nigeria, which is known to be endemic for viral infectious diseases that are recognized etiologic agents of SHL. Specifically, the primary aims of the study were to determine the 1) prevalence of each grade of hearing loss defined by the pure tone average (PTA) as defined in the World Health Organization (WHO) classification and to determine 2) the prevalence of different audiogram patterns in this population. The prevalence of different audiogram patterns may therefore give insight into the potential causes of SHL in this population. The study assessed the prevalence of moderate or higher degree hearing loss at different age groups, with particular attention to the younger population which usually does not have a high prevalence of hearing loss.

.”

b. The time frame of the review requires substantiation, justification, and expansion while accounting for its contemporaneity.

**RESPONSE:** We have made the following amendments to the METHODS section to address the time frame and to add details, as follows.

“This 10-year retrospective cohort study was carried out between October 2010 and September 2020 at the Otolaryngology/Head and Neck Surgery Department of the Jos University Teaching Hospital, Plateau State, Nigeria. The Jos University Teaching Hospital is a tertiary level health center located in North Central Nigeria. It receives referrals from neighboring states of Nasarawa, Bauchi, Benue, Taraba, parts of Kaduna and Kano, with a combined population of over 20 million. [8] The time frame of 10 years chosen for this study as well as the setting of a tertiary hospital was intended to capture as large a portion of the population as possible in order for the findings to have strong external validity and fidelity to the general population in North Central Nigeria. “

c. The data collection describes the assessment of patients, but this manuscript is described as a review so the methodology of the review of the patient data ought to be described i.e. how data were accessed, selected, cleaned, mined, recorded, permissions, by whom, etc. etc.

**RESPONSE:**

We have removed the remark that this study is a “review”.

d. Inclusion and exclusion criteria need to be elucidated.

**RESPONSE:**

We have added inclusion and exclusion criteria in the Methods section as follows.

“The inclusion criteria were all patients who visited the otolaryngology clinic with a chief complaint of hearing loss and also had a pure tone audiogram. The exclusion criteria were patients who had a conductive hearing loss of 15dB or greater or had missing data on audiogram, age or sex.”

e. Consideration needs to be given to the reliability of the PTA as “PTA average was used to assign grades of hearing loss” (page 8) especially since there is no mention of what was considered in cases of big differences between adjacent frequencies, for example.

**RESPONSE:**

We used the same PTA calculation and hearing loss grading based on the method used in the WHO “World report on hearing” in order to allow direct comparison. Cases where the audiometric data differed significantly across frequencies is addressed separately in the section detailing audiogram patterns. The WHO grading system of hearing loss is a validated scoring tool for age-related hearing loss that predicts deficits in communication performance in unaided patients.

f. Please offer a reference for the configurations of the audiograms.

**RESPONSE:**

The reference has been added in the Methods and in the References.

[10] Pittman AL, Stelmachowicz PG. Hearing loss in children and adults: audiometric configuration, asymmetry, and progression. Ear Hear. 2003 Jun;24(3):198-205.

g. It is stated that analysis was conducted on SPSS but the specific analyses are not elucidated, especially with related to descriptive and inferential statistics, especially since in the results, the term ‘trend’ is used as is ‘disproportionately’ while there’s also reference to ‘twice the frequency’ but little detail is provided of that significance.

**RESPONSE:**

Details of the statistical analysis was added in the Methods section as follows.

*Statistical analysis*

The percentages of each audiogram pattern and grade of hearing were separated by sex and age group and compared using Chi-square test using SPSS software Version 23.0. 1989, 2015, 2-sided, P<0.05 was set as significant difference.

Analysis and results were added as Table 2 and Table 3. Text of the Results section was revised as follows.

In age groups 60 and older, the prevalence of patients with moderate or higher grade hearing loss increased significantly, whereas unilateral hearing loss was more frequently seen at ages less than 60 (Table 2). Audiogram patterns showed specific patterns at different age groups. There was significantly higher prevalence of patients with downward sloping patterns in age group 60 and older compared to ages 8 – 29 years old. On the other hand, there was significantly higher prevalence of upward sloping pattern in patients 8 – 29 years compared to age 60 and older. The cookie bite pattern and flat pattern was less prevalent in age group 60 and older (Table 3).

2. RESULTS, DISCUSSION, & CONCLUSION
   a. Reference is made to normal hearing but, although hearing may be within normal limits, there may still be hearing or listening difficulties as acknowledged in the discussion when it is stated, “Any reduction of hearing capacity with disability should be investigated seriously due to its close association with declines in social function.” so please look at replacing ‘normal hearing’ with ‘hearing within normal limits’ as delineated by the analyses.

**RESPONSE:** We have changed the terminology as suggested.

b. The intention is stated as a measurement of burden but the results are reflective of audiometric configurations, degree, etc. but the burden, especially since reference was made to “years lived with disability.”

**RESPONSE:** The authors appreciate the insight on this inconsistency. We were hoping to convey prevalence instead of the burden and have made this amendment. Therefore, the audiometric configurations and degrees of hearing loss were intended to reflect the prevalence of hearing loss.

c. The comments related to the methodology feed into the review of the results and reflect the same need for further exegesis.

**RESPONSE:** The methods section and results sections have been revised as follows.

Methods section:

*Statistical analysis*

The percentages of each audiogram pattern and grade of hearing were separated by sex and age group and compared using Chi-square test using SPSS software Version 23.0. 1989, 2015, 2-sided, P<0.05 was set as significant difference.

Results section:

In age groups 60 and older, the prevalence of patients with moderate or higher grade hearing loss increased significantly, whereas unilateral hearing loss was more frequently seen at ages less than 60 (Table 2). Audiogram patterns showed specific patterns at different age groups. There was significantly higher prevalence of patients with downward sloping patterns in age group 60 and older compared to ages 8 – 29 years old. On the other hand, there was significantly higher prevalence of upward sloping pattern in patients 8 – 29 years compared to age 60 and older. The cookie bite pattern and flat pattern was less prevalent in age group 60 and older (Table 3).

d. The conclusion ought to be strengthened in relation to the aforementioned comments while also delineating conclusions which strongly emanate from the findings and the intention of this project.

**RESPONSE:** The Discussion/Conclusion section was revised. In summary, we emphasized that the patients in our cohort had earlier onset hearing loss and higher prevalence of flat audiogram configuration which might suggest an additional etiology of hearing loss in North Central Nigeria. Lassa virus infection may be a contributor, which is known to cause hearing loss. Please see Discussion/Conclusion section for details.

3. FORMAT & STYLE
   a. Please replace the term ‘subjects’ to refer to the patient records, or records, etc. and patients instead of subjects when referring to people.

**RESPONSE:**

The term subjects was changed to patients.

b. Please replace ‘down sloping’ with ‘downward sloping’ and ‘upsloping’ to ‘upward sloping’

**RESPONSE:**

The terminology was changed as suggested.

c. Please spell out acronyms in full the first time before using the acronym.

**RESPONSE:**

The acronyms were spelled out in full when appearing for the first time in the manuscript text.

d. Please use fewer instead of less where appropriate for items which can be counted e.g. “… and an even lower availability of audiologists with less than 1 per million…” amongst others.

**RESPONSE:**

This was revised in the manuscript as suggested.

e. Please use consistent terminology e.g. ENT and otolaryngologist if it applies (since technically otolaryngologist is not synonymous with ENT but otorhinolaryngologist would be a closer match).

**RESPONSE:**

Changes have been made to unify the term to “otolaryngologist”

f. Please use lower case letters for common nouns e.g. an Otolaryngology Clinic

**RESPONSE:**

This was changed as suggested.

g. Please check for typographical errors e.g. Sentiero//ADVACED ought to be ADVANCED.

**RESPONSE:**

Incidences of the above have been changed as suggested.

h. The word ‘data’ is a plural noun so needs to be accompanied by a plural verb.

**RESPONSE:**

Incidences of the above have been changed as suggested.

**REVIEWER #2:**

1. The title of the manuscript is misleading as no information was presented regarding the medical history (specifically related to Lassa Fever). The manuscript only addressed sensorineural hearing loss, thus discarding the information on conductive hearing loss. This (SNHL) should be reflected in the title.

**RESPONSE:**

The title has been revised as “Analysis of sensorineural hearing loss in patients attending an otolaryngology clinic in North Central Nigeria”

2. Significant editorial care (typographical and grammatical) is required. The consistent use of terminology is encouraged (degree/grade; ENT/otolaryngologist, etc.). Although standard English is used, it is sometimes difficult to understand - have the manuscript professional edited before the resubmission.

**RESPONSE:** We have done our best to improve editorial care throughout the entire manuscript as suggested.

3. If the authors decide to change the title, the introduction section should also be amended. A suggestion is to start with information presented from lines 54- 67 and then highlight the potential contributing factors to hearing loss such as Lassa Fever, CMV, presbycusis, etc. in North Central Nigeria.

**RESPONSE:** We appreciate the suggestions. We have made major revisions to the Abstract and Introduction sections. Please see Abstract and Introduction for details.

4. Methodology: The lack of more demographic information (e.g., medical history on potential cause of hearing loss) is a limitation of this study. Further, only descriptive statistics were used, would the use on inferential statistics not strengthen the findings?

**RESPONSE:** We appreciate the input. We have added a statement in the discussion to address the limitations of our study.

We have added statistical analysis, detailed in Table 2 and Table 3 to help strengthen the findings and added texts associated with these analyses in the methods, results, and discussions sections.

DISCUSSION

“The lack of other demographic information such as medical and social history results in difficulty associating etiologies with hearing loss especially if patients have multiple possible causative factors. This was a limitation in our study”.

*Statistical analysis*

The percentages of each audiogram pattern and grade of hearing were separated by sex and age group and compared using Chi-square test using SPSS software Version 23.0. 1989, 2015, 2-sided, P<0.05 was set as significant difference.

RESULTS

In age groups 60 and older, the prevalence of patients with moderate or higher grade hearing loss increased significantly, whereas unilateral hearing loss was more frequently seen at ages less than 60 (Table 2). Audiogram patterns showed specific patterns at different age groups. There was significantly higher prevalence of patients with downward sloping patterns in age group 60 and older compared to ages 8 – 29 years old. On the other hand, there was significantly higher prevalence of upward sloping pattern in patients 8 – 29 years compared to age 60 and older. The cookie bite pattern and flat pattern was less prevalent in age group 60 and older (Table 3).

5. The discussion of the findings can be more robust and contextually relevant and should be amended based on the decision made by the authors regarding the title, etc.

**RESPONSE:** The Discussion/Conclusion section was revised. In summary, we emphasized that the patients in our cohort had earlier onset hearing loss and higher prevalence of flat audiogram configuration which might suggest an additional etiology of hearing loss in North Central Nigeria. Lassa virus infection may be a contributor, which is known to cause hearing loss. Please see Discussion/Conclusion section for details.

Sincerely,

**Nathan Shehu, MD**

Medicine Department, Jos University Teaching Hospital

Address: Lamingo Street, Jos, Plateau State, Nigeria 930105

Email: [nyshehu@yahoo.com](mailto:nyshehu@yahoo.com)
