# Supplementary material for: Analysis of sensorineural hearing loss in patients attending an otolaryngology clinic in North Central Nigeria
Source: PLOS Glob Public Health. 2023 Apr 25;3(4):e0000685. doi: 10.1371/journal.pgph.0000685 (PMC10128921; doi:10.1371/journal.pgph.0000685)
Supplement: Attachment — Submitted filename: Nigeria Audiogram_PLOS_ Response to Reviewers.docx [file pgph.0000685.s003.docx]

January 29, 2023

Dear Dr. Kyobutungi & Pai,

Thank you for inviting our team to submit a revision of our manuscript entitled, "A ten-year retrospective review of audiogram configurations of patients attending an otolaryngology clinic in Lassa fever endemic North Central Nigeria. '' to the *PLOS Global Public Health* Journal. We appreciate the effort and time that the reviewers dedicated in providing their insight and helpful feedback. It is with our great pleasure that we are resubmitting our article for further consideration. We incorporated the changes from the insightful suggestions that were provided. We hope that our edits and responses address and satisfy the concerns you and the reviewers have provided.

To aid in the facilitation of your review of the revisions, the following is a point-by-point response to the questions and comments.

**REVIEWER #2 SUGGESTIONS:**

1. The objective stated in abstract does not correspond to the aims outlined in the introduction

**RESPONSE:** The authors appreciate identifying inconsistencies in our objectives for the project. We have made the following amendments in the abstract to address this.

“The objective of this study was to estimate period prevalence of hearing loss and audiogram patterns of patients attending an otolaryngology clinic in North Central Nigeria .”

2. Integrate into the previous sentence. E.g., a 10-year retrospective cohort study was carried out analyzing 1507 patient records....

**RESPONSE:** We have made the following amendment to address this suggestion.

“A 10-year retrospective cohort study was carried out analyzing 1507 patient records of pure tone audiograms of patients at the otolaryngology clinic at Jos University Teaching Hospital, Plateau State, Nigeria.”

3. “The Flat audiogram configuration was the most prevalent in younger age groups. The downward sloping configuration was the most prevalent in the 60 and older age groups, while the prevalence of upward sloping configuration declined with age.” Consider removing these sentences

**RESPONSE:** These sentences have been removed.

4. Consider adding the prevalence rate here “Compared to other studies, there was a higher prevalence of overall sensorineural hearing loss and higher proportions of the flat audiogram configuration among the younger age patients”

**RESPONSE:** We have amended the following to address this suggestion.

“Compared to other studies, there was a higher prevalence of overall sensorineural hearing loss (24-28% in our study compared to 1.7 -8.4% globally) and higher proportions of the flat audiogram configuration among the younger age patients (40% in younger patients compared to 20% in patients older than 60 years).

5. “We hope our study will be the basis for additional studies to determine the cause of the hearing loss in North Central Nigeria.” Consider removing the sentence.

**RESPONSE:** We have removed the sentence above.

6. Please reorganize the references. Number according to where they appear in the text – start with 1…

**RESPONSE:** The references have been organized appropriately and accurately throughout the entirety of the manuscript.

7. Use this acronym (WRH) throughout the report. There are a number of instances that should be edited.

**RESPONSE:** We went through the manuscript to ensure addition of the acronym where appropriate.

8. “Specifically, the primary aims of the study were to determine the 1) prevalence of each grade of hearing loss defined by the pure tone average (PTA) as defined in the World Health Organization (WHO) classification and to determine 2) the prevalence of different audiogram patterns in this population. Audiogram patterns are associated with certain etiologies [10]. Consider integrating into the materials and methods.

**RESPONSE:** We have integrated sentences in the methods as suggested.

“The primary aims of the study were to determine the 1) prevalence of each grade of hearing loss defined by the pure tone average (PTA) as defined in the World Health Organization (WHO) classification and to determine 2) the prevalence of different audiogram patterns in this population. Audiogram patterns are associated with certain etiologies [9].”

9. Page 5. Materials and methods. Consider using “gender” rather than “sex”

**RESPONSE:** The authors have made this change throughout the manuscript.

10. Page 5. Materials and methods. Demographic data ---- such as?

**RESPONSE:** We have omitted this reference about demographic data to avoid confusion.

11. “The prevalence of different audiogram patterns may therefore give insight into the potential causes of SHL in this population. The study assessed the prevalence of moderate or higher degree hearing loss at different age groups, with particular attention to the younger population which usually does not have a high prevalence of hearing loss.” Relevance? Inappropriately placed.

**RESPONSE:** The authors decided to remove this sentences given its questionable relevance for the manuscript. We appreciate the insight.

12. Integrate with previous sentence. E.g., The percentage of patients with each grade of hearing loss was calculated and categorised into the following age groups...

**RESPONSE:** The following sentences below have been integrated as one sentence.

“The percentage of patients with each grade of hearing loss was calculated and categorized into the following age groups: 8-19 years, 20-29 years, 30-39 years, 40-49 years, 50-59 years, 60-69 years, 70-79 years, 80-90 years, and 90+ years.”

13. “The percentages of each audiogram pattern and grade of hearing were separated by sex and age group and compared using Chi-square test using SPSS software Version 23.0. 1989, 2015, 2 sided, P<0.05 was set as significant difference” Relevance? The descriptive statistics are used are more than adequate.

**RESPONSE:** The authors appreciate the feedback on the description of our statistics. We implemented a better explanation of the descriptive statistics utilized.

“Statistical analysis was conducted using SPSS Statistics. The percentages of each audiogram pattern and grade of hearing were separated by sex and age group. Association between these variables was analyzed using a chi-square test which revealed a statistically significant difference (*X* ^2^ =136.25, p<0.05, 2-sided).

14. “Between the ages of 8-49 years old, the majority of the patients had hearing within normal limits: 49% at 8-19 years, 52% at 20-29 years, 48% at 30-39years, and 41% at 40-49 years age groups respectively. From 50-69 years old, mild hearing loss was the most prevalent grade of hearing loss: 38% at 50-59 years and 35% at 60-69 years age groups respectively.” Redundant to, in the text, to repeat findings. Only highlight the most pertinent findings in the text.

**RESPONSE:** The authors appreciate careful attention to our results section. We have corrected the description to only describe pertinent findings as suggested.

“Between the ages of 8-49 years old, the majority of the patients had hearing within normal limits with over half (52%) in the 20-29 year-old group. From 50-69 years old, mild hearing loss was the most prevalent grade of hearing loss, most notably making up 38% in 50-59 year-old patients.”

15. “The WHO grading system of hearing loss is a validated scoring tool for age-related hearing loss that predicts deficits in communication performance in unaided patients [17].” This should be moved to the appropriate place in the materials and methods section. Not well placed here.

**RESPONSE:** The authors have moved this sentence to the materials and methods section as suggested.

16. Regarding percentage column on Table 1 - of sample? Make it more explicit.

**RESPONSE:** The authors changed the column to be titled “Percentage of Sample.”

17. Regarding the Tables - The N should not be capitalized - in all instances use the lower case 'n'

**RESPONSE:** These changes have been addressed and amended throughout the tables.

Sincerely,

**Nathan Shehu, MD**

Medicine Department, Jos University Teaching Hospital

Address: Lamingo Street, Jos, Plateau State, Nigeria 930105

Email: [nyshehu@yahoo.com](mailto:nyshehu@yahoo.com)
